# Climate change accelerates growth of urban trees in metropolises worldwide

**DOI:** 10.1038/s41598-017-14831-w

**Published:** 2017-11-13

**Authors:** Hans Pretzsch, Peter Biber, Enno Uhl, Jens Dahlhausen, Gerhard Schütze, Diana Perkins, Thomas Rötzer, Juan Caldentey, Takayoshi Koike, Tran van Con, Aurélia Chavanne, Ben du Toit, Keith Foster, Barry Lefer

**Affiliations:** 1 Chair for Forest Growth and Yield Science, Center of Life and Food Sciences Weihenstephan, Technische Universität München, Hans-Carl-von-Carlowitz-Platz 2, 85354 Freising, Germany; 2 Departamento de Silvicultura y Conservación de la Naturaleza, Universidad de Chile, Santiago de Chile, Chile; 3 Department of Forest Science, Hokkaido University, Sapporo, 060-8589 Japan; 4 Vietnamese Academy of Forest Sciences, Dong Ngac Commune, Tu Liem District, Hanoi, Vietnam; 5 DEVE–Mairie de Paris; Rond-Point de la Pyramide, 75012 Paris, France; 6 Department of Forest and Wood Science, Faculty of AgriSciences, Stellenbosch University, Private BagX1, Matieland, 7602, South Africa; 7 Brisbane City Hall, 64 Adelaide St, Brisbane, QLD 4000 Australia; 8 Department of Earth and Atmospheric Sciences, University of Houston, 4800 Calhoun Road, 312 Science & Research Bldg 1, Houston, TX 77204-5007 USA

## Abstract

Despite the importance of urban trees, their growth reaction to climate change and to the urban heat island effect has not yet been investigated with an international scope. While we are well informed about forest growth under recent conditions, it is unclear if this knowledge can be simply transferred to urban environments. Based on tree ring analyses in ten metropolises worldwide, we show that, in general, urban trees have undergone accelerated growth since the 1960s. In addition, urban trees tend to grow more quickly than their counterparts in the rural surroundings. However, our analysis shows that climate change seems to enhance the growth of rural trees more than that of urban trees. The benefits of growing in an urban environment seem to outweigh known negative effects, however, accelerated growth may also mean more rapid ageing and shortened lifetime. Thus, city planners should adapt to the changed dynamics in order to secure the ecosystem services provided by urban trees.

## Introduction

Numerous studies show substantial effects of climate change on plant growth^1–5^. The combination of increasing temperatures, extended growing seasons and reduced or intra-annually redistributed precipitation can increase tree growth in boreal and temperate climate zones, often in higher altitudes^5^. The same factors, however, reduce growth in warmer and drier zones where plant growth becomes water limited due to these changes^6,7^. The latter may apply particularly to plants in city centres where the urban heat island effect can aggravate this limitation^8–10^. The average annual daytime surface urban heat island effect (mean of 419 large cities) is 1.5 ± 1.2 °C^11^. Other studies report that the air temperature in urban areas may be as much as 2°–10 °C higher than in the surrounding nonurban areas^12^. These warmer temperatures may substantially affect the living conditions of plants, particularly if precipitation patterns change simultaneously^12^. Therefore, many studies address how climate change, on top of the heat island effect, modifies the manifold functions and services provided by urban plants – especially urban trees – as the most prominent and long-lived elements of urban ecosystems. These previous studies are mainly focused on how climate change alters urban species composition^9^, carbon storage^13,14^, and biodiversity^15^. These studies were primarily based on model predictions and investigated various adaptation measures^7,16^ such as choice of drought resistant species and various environmental provenances^17,18^. However, less is known about how climate change and the urban heat island in combination affect the vitality and growth of urban plants. While the impacts of climate change on tree growth have been extensively studied in forests^19–21^, only limited information is available for urban environments^22^.

**Figure 1:**
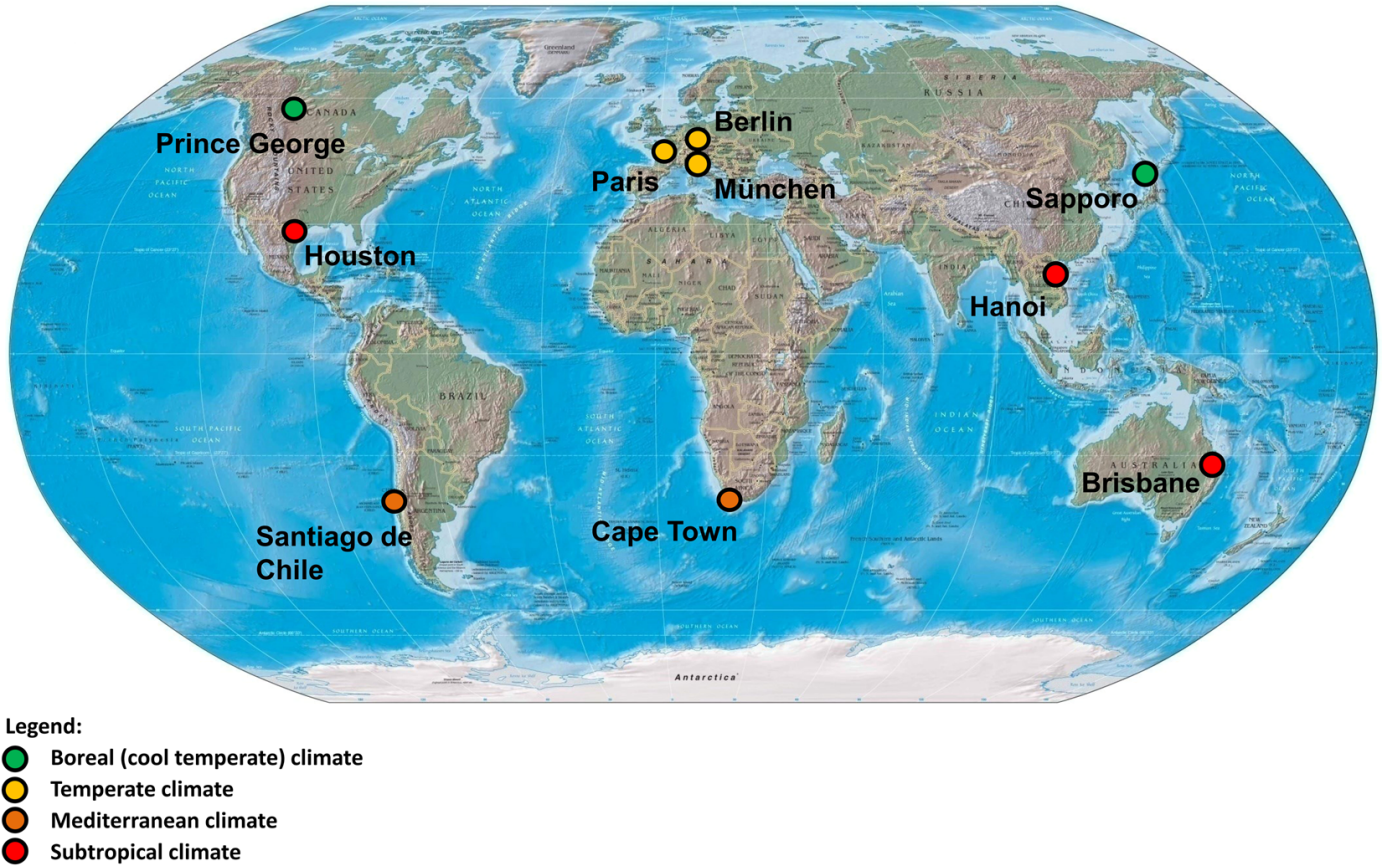
Metropolises, where trees were sampled for this study. (Map was created by modifying the open access file: Physical map of the world, April 2001 (3856492622).jpg [URL: https://commons.wikimedia.org/wiki/File%3APhysical_map_of_the_world%2C_April_2001_(3856492622).jpg (downloaded Dec. 2013)], author: http://maps.bpl.org, licensed under: https://creativecommons.org/licenses/by/2.0/)

This study analyzes urban tree growth under climate change based on a worldwide increment core sampling and dendrometric tree ring analysis, dating back 150 years. In ten metropolises in boreal, temperate, Mediterranean, and subtropical climate (Fig. 1, Table 1, Figure S1) we sampled a total of 1383 mostly mature trees (see Table 2 for sub-sample sizes) using dendrometric measurements and by taking increment cores for dendrochronological analyses. In each city we selected one common species which occurred from the urban centre to the rural outskirts (Figure S1). The rationale behind this selection was to obtain an overarching collection of species which typically thrive under the respective site and climate conditions, and to obtain sufficient samples of trees both suffering and not suffering from the urban heat island effect. Only trees showing no visible signs of damage or disease were taken into account for sampling (see Methods section for a detailed description of the sampling procedure).

**Table 1:** Geography and climate for the metropolises included in this study.

City	Country	Geographic Position	Altitude above Sea Level [m]	Mean Annual Precipitation 1981–2010 [mm a^−1^]	Mean Annual Temperature 1981–2010 [°C]	Climate Zone
Sapporo	Japan	43.07°N 141.34°E	17	1109	8.9	Boreal (Dfb)
Prince George	Canada	53.55°N 122.45°E	691	594	4.3	Boreal (Dfc)
Berlin	Germany	52.31°N 13.24°E	51	591	9.5	Temperate (Cfb)
Munich	Germany	48.14°N 11.58°E	515	948	9.7	Temperate (Cfb)
Paris	France	48.51°N 2.21°E	65	632	12.3	Temperate (Cfb)
Santiago de Chile	Chile	33.27°S 70.40°W	520	325	14.7	Mediterra-nean (Csb)
Cape Town	South Africa	33.55°S 18.25°E	44	544	16.7	Mediterra- nean (Csb)
Hanoi	Vietnam	21.2°N 105.51°E	19	1597	24.6	Subtropical (Cwa)
Brisbane	Australia	27.28°S 153.2 E	6	1076	20.3	Subtropical (Cfa)
Houston	USA	29.46°N 95.23°W	29	1091	21.0	Subtropical (Cfa)

The abbreviated climate zone notations refer to the Köppen-Geiger climate classification^60^.

**Table 2:** Characteristics of the sampled trees with mean, minimum and maximum of measured tree sizes.

City (sampling year)	Species	Number of Sampled Trees (rural, urban)	Diameter in Breast Height [cm]	Tree Height [m]	Height to Crown Base [m]	Crown Projection Area [m²]
Sapporo (2012)	*Abies sachalinensis* Mast.	103 (45, 58)	33.4 (20.0–77.5)	17.5 (11.3–32.0)	6.3 (2.0–18.5)	28.4 (4.6–148.9)
Prince George (2012)	*Picea glauca* (Moench) Voss	109 (20, 89)	40.6 (27.7–56.5)	27.5 (17.7–36.8)	7.2 (1.8–16.6)	22.7 (4.8–61.2)
Berlin(2010-2013)	*Tilia cordata* Mill.	252 (107, 145)	44.2 (16.5–81.1)	16.9 (8.1–29.1)	4.7 (1.8–15.1)	82.3 (19.8–286.4)
Munich (2013)	*Aesculus hippocastanum* L.	193 (28, 165)	63.3 (19.6–117.0)	16.1 (7.4–27.2)	3.3 (0.5–9.7)	99.4 (25.6–256)
Paris (2013)	*Platanus x hispanica* Münchh.	133 (30, 103)	64.8 (40.3–144.0)	18.8 (6.8–34.5)	4.7 (2.5–10)	147.5 (23–648.5)
Santiago de Chile (2012)	*Robinia pseudoacacia* L.	129 (30, 99)	41.4 (19.8–56.1)	15.3 (4.8–31.5)	2.7 (1.7–6.3)	14.6 (1.5–49.0)
Cape Town (2011)	*Quercur robur* L.	69 (21, 48)	67.9 (40.3–112.9)	15.6 (9.7–22.8)	3.7 (2.1–7.3)	168.2 (56.9–341.7)
Hanoi (2012)	*Khaya senegalensis* (Desr.) A.Juss.	149 (56, 93)	73.4 (44.1–123.1)	22.6 (14.1–36.0)	5.6 (2.2–10.7)	136.9 (31.0–421.5)
Brisbane (2013)	*Araucaria cunninghamii* Aiton ex. D.Don	66 (3, 63)	40.7 (15.7–129.5)	17.3 (3.1–33.5)	3.2 (0.6–7.1)	45.5 (8.4–422.8)
Houston (2014)	*Quercus nigra* L.	180 (49, 131)	59.9 (34.2–98.0)	16.2 (10–25)	3.8 (1.2–11.6)	162.6 (37–442)

Stem diameter at breast height refers to a measurement height of 1.3 m.

The following species were covered by the study: *Abies sachalinensis* Mast. (Sachalin fir), *Picea glauca* (Moench) Voss (white spruce), *Tilia cordata* Mill. (small-leaved lime), *Aesculus hippocastanum* L. (horse-chestnut), *Platanus x hispanica* Münchh. (London plane), *Robinia pseudoacacia* L. (black locust tree), *Quercus robur* L. (English oak), *Khaya senegalensis* (Desr.) A.Juss. (African mahogany), *Araucaria cunninghamii* Aiton ex. D.Don) (hoop pine), and *Quercus nigra* L. (water oak). See Table 2 for city attribution and characteristic tree dimensions.

We used these data to scrutinize whether tree growth, as expressed by the relationship between tree age and size, differs between urban and rural trees and between recent and past climate conditions in general, and whether such effects differ across climate zones. We chose to evaluate basal area (cross-sectional stem area at a height of 1.3 m) as the tree size variable of interest on the basis that its growth can be straightforwardly reconstructed from increment cores and in light of the fact that it is closely linked to a tree’s biological production^23^.

## Results

The equation $\mathrm{ln}(ba)=a+b\,\cdot \,\mathrm{ln}(age)$ with *ba* being a tree’s basal area was well applicable for describing the size growth of individual trees. Based on linear mixed models, we tested for differences in the parameters *a* and *b* related to (i) whether an observation was from before or since 1960, (ii) whether a tree belonged to the urban or the rural zone, and (iii) the climate zone (boreal, temperate, Mediterranean, subtropical) with which a tree is affiliated. The time period distinction under i was chosen in analogy to recent studies which show evident climate change effects on forest growth since about the 1960s^5,24,25^. See methods section for a detailed method description.

**Figure 2:**
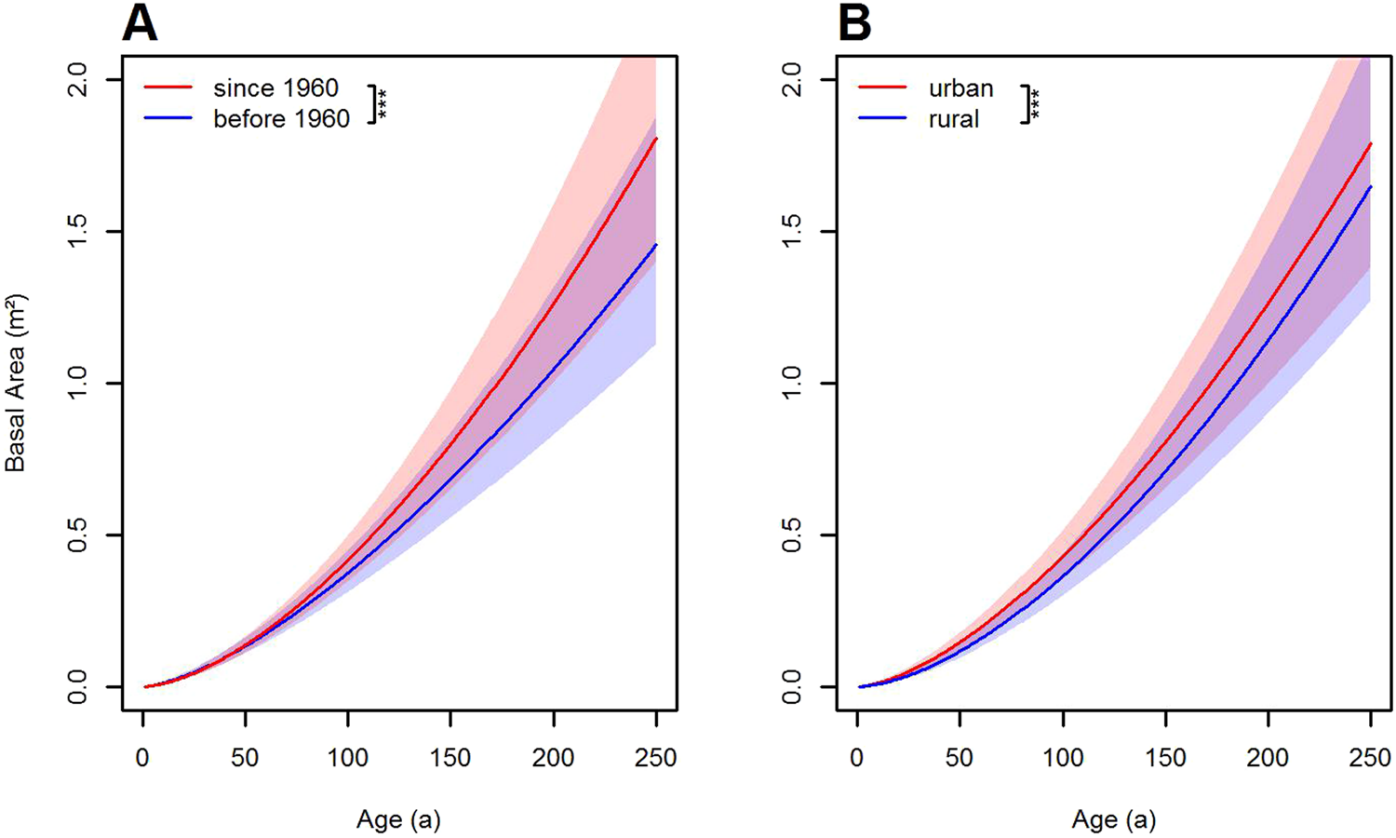
Effect of climate change and urban zone on tree size growth across all climate zones. (**A**) Expected basal area growth of urban and rural trees together, before and since 1960, (**B**) Expected basal area growth of urban compared to rural trees. Shaded bands visualize the prediction standard error of the curves. Despite the broad overlap of these bands, the curves in both diagrams differ in both parameters with a significance level of p < 0.001 (bracket with symbol ‘***’ in the diagram legends, see Tables S1 and S2, equations 3 and 4).

First, without differentiating between urban and rural trees and between regions, we checked for an overarching difference in tree growth in the periods before and after 1960. This difference proved to be significant (Fig. 2A, Table S1, Equation 3). On average, tree growth since 1960 has occurred more rapidly than before. However, while the relative difference in tree size is negligible up to an age of about 50 years (+2%), it increases with age (+11% and +17% at ages 100 and 150 years, respectively). A second global test without any period and region distinction showed that, on average, urban trees can be expected to reach a greater size than rural trees of the same age (Fig. 2B, Table S2, Equation 4). In a closer view, the relative size difference of urban compared to rural trees at the same age declines with increasing age. While it amounts to about 25% at an age of 50 years, it reduces to 18% at 100 years and to 14% at 150 years.

**Figure 3:**
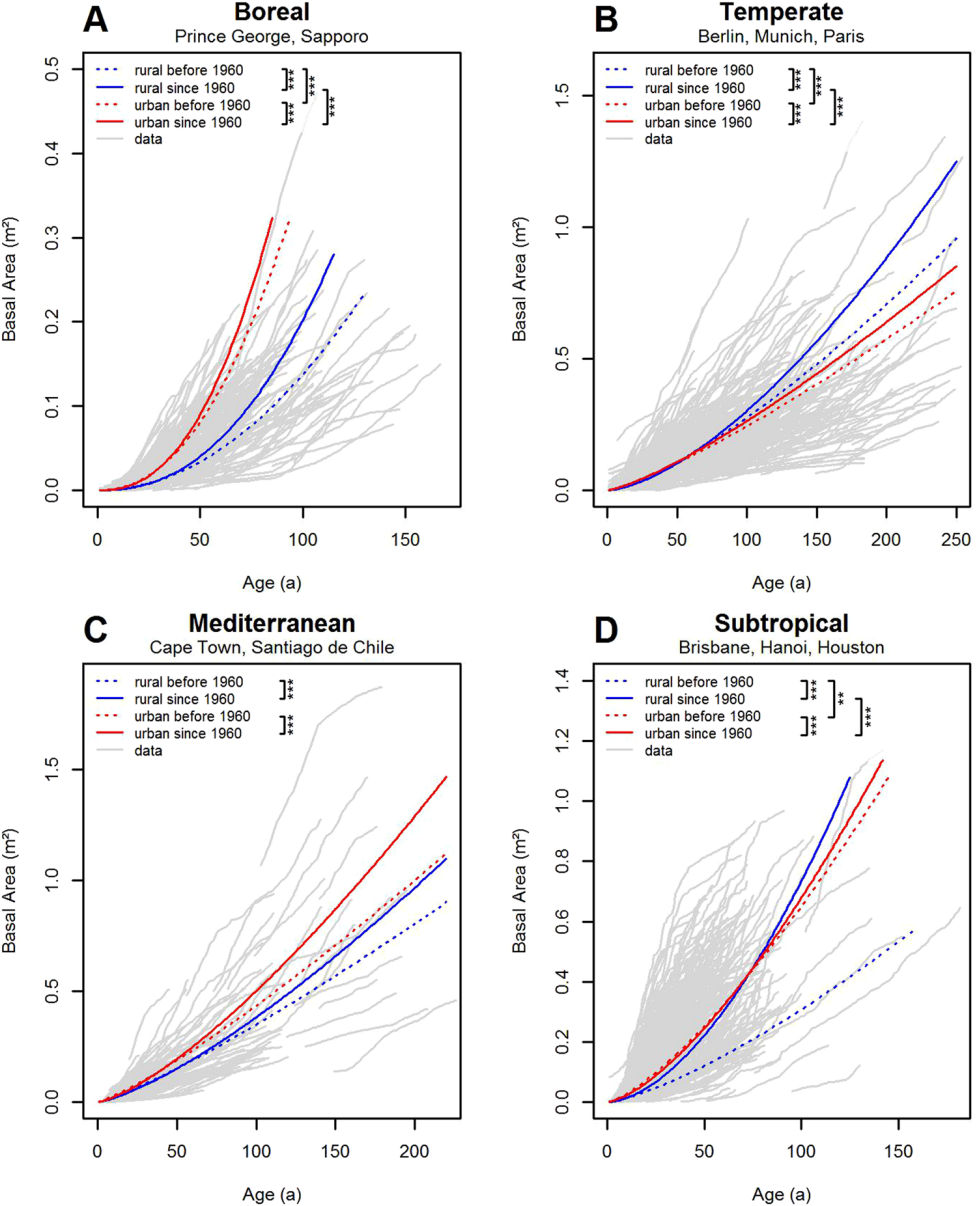
Effect of urban zone and climate change on tree size growth by climate zones (**A** boreal, **B** temperate, **C** Mediterranean, **D** subtropical). Significant differences between two curves are indicated by brackets connecting the corresponding legend entries and showing the level of significance (**p < 0.01, ***p < 0.001, see Table S3, equation 5, Tables S4A–D).

More detailed analyses, differentiating between the four climate zones, confirm these general results but also reveal interesting peculiarities (Fig. 3, Table S3, Equation 5). In the boreal zone (Fig. 3A, Table S4A), both before and after 1960, urban trees showed a very strong superiority in growth compared to their rural counterparts. In both, urban and rural zones, trees have grown significantly faster since 1960 than before. Before 1960, subtropical urban trees (Fig. 3D, Table S4D) grew significantly faster than rural trees; after 1960, the rural trees reached about the same level of the age-size relationship as urban trees had before. Although the growth curve of urban trees also changed significantly after 1960, this change is not relevant due to its minor magnitude. For the Mediterranean climate zone, no significant growth differences between urban and rural trees could be detected, either before or after 1960 (Fig. 3C, Table S4C), while for both urban zones there is a clear growth acceleration from before 1960 to the subsequent period. The temperate climate zone (Fig. 3 B, Table S4B) was the only case we found where urban trees grew significantly slower than rural trees. This holds true for both periods, before and since 1960.

In summary, when broken down to the level of climate zones, the general trend of growth acceleration since the 1960s seems to be due to an overarching effect, while the urban zone effect is slightly more heterogeneous.

## Discussion

Urbanization is one of the 21st century’s megatrends. Based on UN calculations, the urban population will increase by more than 60% by 2030 and continue to near 70% by 2050^25^. In this context, urban trees and their crucial role for public health and quality of life are highly valued. With this study we want to contribute to the understanding of urban tree growth. While we can document clear growth effects based on an unusually broad dataset and solid statistical procedures, this work is not a mechanistic analysis about the causes behind the reported trends. However, among other points, we try to identify probable reasons from the existing body of literature in the following discussion. Moreover, we hope our results will trigger mechanistic studies in order to gain a deeper understanding of the physiological processes underlying our observations.

### Environmental change effects on urban and near-urban rural trees

We show that climate change over the last century has been accompanied by higher growth rates of urban and nearby rural trees since 1960. This observed accelerated growth reflects a pattern that has also recently been reported for forest trees. Kauppi *et al*.^26^ identified an increased tree and stand growth in boreal forests, Fang *et al*.^27^ found a similar pattern in Japanese forests and Pretzsch *et al*.^5^ revealed similar results in temperate forests in Central Europe. The observed growth acceleration of urban trees (14%–25%) is similar to the findings related to forests and occurred to some extent also in agricultural systems^28,29^. Obviously, there have been changes in environmental conditions fostering a generally accelerated tree growth regardless of climate zone and land classification. In this context, global warming^30^, going along with extended growing seasons^31^, higher atmospheric CO_2_-concentrations^30,32,33^, fertilization through N-deposition^32^ and diurnal temperature range^34^ are discussed as possible driving forces. Despite possible negative effects of global climate change on tree growth – such as drought events which may reduce tree and stand growth^21,35,36^ or even cause a die off^37–39^ – the observed trees seemed to have benefitted so far. This happened in a remarkably uniform way: Both, urban and rural trees along all investigated climate zones significantly accelerated their growth in the last decades.

### Urban vs. rural tree growth

Urban trees in the boreal zone grew faster than their rural counterparts both before and after 1960. A similar urban tree growth response was observed in the subtropical zone, but only after 1960.

The higher growth rates of urban trees (as compared to rural trees) seem to be closely related to the urban climate which is characterized by the urban heat island effect, leading to an increase of the daytime surface and air temperature of sealed city centers by up to 3 and 10 °C, respectively^11,12,40^. The urban heat island involves higher temperatures in cities compared to the surrounding landscapes that may stimulate photosynthetic activity if the temperature optimum of a species is not yet reached^41^ and extend the growing season^31^ by up to 8.8 days per year^42,43^. Numerous studies show an advanced onset of phenological phases in urban areas compared to their rural surroundings^44,45^. Higher CO_2_ concentrations^33,46,47^, larger annual atmospheric N-deposition^46^ and lower ozone concentration^48^ in urban areas compared to their rural surroundings^48^ might further foster urban tree growth. Particularly in the cities located in the boreal climate zone, urban trees showed higher productivity than rural trees. Because of the high precipitation in these climates and thus non-limited water supply for the trees, the above-mentioned influences of increased temperature and longer growing seasons, higher CO_2_ concentrations and N-deposition might be effectively accelerating urban tree growth.

However, we did not only observe such superior growth of urban zone trees. Under a Mediterranean climate we found no significant difference between urban and rural tree growth, neither before, nor after 1960. And in contrast to other regions, temperate zone, urban zone trees grew significantly less than rural ones, both before and after 1960. While adverse and beneficial effects of rural and urban zones seem to cancel out under Mediterranean conditions, the adverse urban zone effects seem to constrain tree growth in temperate climate cities. Urban trees can suffer from substantial water stress due to high temperatures, modified precipitation patterns, and unfavorable soil conditions due to impervious surfaces and compacted soils in urban areas^49^. Along with mechanical impacts^50^ and reduced gas diffusion within the rhizosphere^51^, these effects may reduce root growth and in turn hamper a tree’s water uptake. We assume that the trend towards a declining difference in growth rates between urban and rural trees with increasing age is closely linked to limited water supply of bigger trees. The higher potential water consumption of old (big) trees compared to young (small) ones cannot be fulfilled under urban conditions and this results in reduced tree growth.

### Urban zone and environmental change effects

As reported above, environmental changes since the 1960s resulted in consistent growth acceleration of urban and rural trees throughout the investigated climate zones. This occurrence in three of the four climate zones does not change the previous ranking of the urban zones (urban vs. rural) in terms of growth velocity. The results were only different for the subtropical zone, where rural trees showed enormous growth acceleration in contrast to urban trees, where only a minor increase was observed. Overall, the combine trend was a non-relevant difference in urban vs. rural growth rates since the 1960.

If the urban environment can be considered a preview of future climate conditions for nearby rural areas (e.g. warmer and drier), our results suggest that rural trees in subtropical regions will be the first of the non-urban areas to encounter conditions were tree growth rates will decline due to climate change. While such a pattern was not detected for the other investigated climate zones, urban tree growth may very well develop in different directions depending on the various combination of the key causal effects (temperature, water supply, growing season length, CO_2_ and O_3_concentration, N-deposition), their limitations and/or levels as altered by climate change and differing between urban and rural areas^34^. For example, the extension of the growing season length caused by both the urban heat island effect and climate change may be in the magnitude of up to 11% for European cities assuming an urban heat island effect of 8.8 days^42^, a global warming effect of 10.5 days within a period of 30 years^31^ and an average growing season length of 180 days^52^.

Again, adverse conditions in cities like limited rooting space or higher pollution through particulate matter do not seem to cancel out to the benefits of current urban climate and atmospheric conditions for tree growth. However, in the temperate and in the subtropical zone, urban compared to rural tree growth has profited less from the changes in recent decades. This might be seen as a sign that the formerly beneficial urban climate may turn into disadvantageous dry conditions that reduce growth, especially in water-limited climate zones such as subtropics.

### Generalizability

To our knowledge, this is the first study providing an international synopsis about the effects of global climate change and the urban zone on tree growth in cities. As it was obviously impossible to sample the same tree species in all metropolises, one might argue that cross-city analyses were not meaningful due to a lack of comparability. However, given the goals of our study, having a distinct species at each location that is, as indicated by its frequent occurrence, well adapted to past long-term local conditions is both the most realistic and the most preferable option. In this way, the relevance of our results for management is secure and we can safely use past growth of well-adapted trees as a reference. Against this backdrop, it was important to prevent species-specific scaling characteristics from introducing bias into the results reported above. This was achieved by including city-/species-specific random effects on both generic parameters of the relationship between tree age and basal area in all regression models which formed the statistical backbone of our analysis (see Equations 3, 4 and 5).

As this study focuses on the growth of trees, visibly damaged or diseased individuals were excluded from sampling. Thus, our results do not allow statements e.g. about the potentially differing risks of diseases or premature death faced by urban and rural trees. With this work we emphasize the potential of urban trees for bio-monitoring, especially in retrospect. Using tree ring patterns as a source of information about environmental changes we can show the vast footprint of humans on urban tree growth. Both global climate change and the urban heat island effect are reflected in the tree ring patterns. Together these effects accelerate tree growth by an average of 35%, consisting of a global climate change effect of 21% and an urban heat island effect of 14%. We sampled tree species which are i) growing in their optimum in the respective climate zones ii) commonly established in urban areas, and iii) well adapted to the respective (past) climate. Other species which are less adapted may benefit less from the changing climate or suffer more from future developments in the global and urban climates. But interestingly, although the sampled tree species differ in their general traits (e.g. shade or drought tolerance, hydric behavior) an overarching trend in growth shift was observed.

### Consequences

The shown acceleration of tree size growth means increased C sequestration, accelerated spatial above- and below-ground expansion, and earlier provision of many ecosystem services. However, it also means more rapid tree aging, possibly indicating a need for earlier replacement and replanting. In order to sustain the green urban infrastructure, planning and management should adapt to this changing tree growth rate. Whether the accelerated tree growth lowers the mechanical stability, biotic resistance, or safety hazard of urban trees is a topic of ongoing research based on the worldwide network of urban trees established within this study.

## Methods

### Materials

For this study we selected ten cities worldwide distributed over four different climate zones, namely boreal, temperate, Mediterranean, and subtropical (Fig. 1, Table 1). A decisive selection criterion was the permission from the municipal authorities to measure and core trees. Based on the tree registers of the municipal administrations and our own local inventories we selected the most frequent tree species of each city that occurred from the city centre to the suburban and the rural area surrounding the city (Table 2). Our ideal sampling design was to measure and take increment cores of trees along transects from the centre to the rural area of the cities in all four main directions: north, east, south, and west. Along those four transects, the trees of a given species and size should ideally have been selected at distances of 500–1000 m. In many cases, however, we had to deviate from this idealistic design as trees were missing because of building density, lakes and rivers, or specific features like the Table Mountain without oak trees in Cape Town. See Figure S1 for the city-specific sampling arrangements.

We only sampled trees without visible biotic or abiotic damage in order to differentiate general growth trends from individual disease or damage. All trees were dominant and growing more or less solitarily without direct competition from neighbours. The target size range was 30–70 cm diameter at breast height. However, in order to avoid larger gaps along the transects we also included smaller and larger trees. Despite these exceptions, the mean diameter over all sampled trees was 54 cm and about 73% of the trees lay within the target size range mentioned above.

In each city we started the sampling in the urban centre, and proceeded to the suburban and rural zones. We divided the distance from the centre to the urban, suburban, and rural zones for further sampling and tried to get a third of the total sample in each zone. Because of the varying extension of the selected cities, the sample size varies between 252 trees in Berlin and 66 trees in Brisbane. For our purpose we defined the urban zone of a city as the areas which have been under roof for the longest time. Typically, in this zone the soil is totally sealed and the buildings serve mainly business purposes. As suburban zones we selected areas surrounding the urban zone which are continuously covered by buildings but which are mainly places of residence. Typically, soil sealing is slightly lower in these areas than in the actual urban zone. The rural zone was defined as a habitat surrounding the suburban zone with only sparse, at most village-like structures where trees could grow virtually without the direct influence of buildings and soil sealing. Following this procedure, we sampled a total of 1383 trees comprising 348 (25%) urban, 619 (45%) suburban, and 416 (30%) rural trees. As preliminary analyses showed no significant differences between the growth of urban and suburban trees, we did not distinguish between these groups in the further analysis. These analyses were performed with linear mixed models in a very similar way as described below. Thus, in the following text the term “urban” refers to trees from both the original urban and suburban zones and these together are then contrasted with rural trees in the analyses below.

### Methods

As the most important tree property for our purpose we measured each tree’s stem diameter at breast height (*dbh*), which means a measurement height of 1.3 m, with a girth tape. In addition, total tree height and height to crown base (*hcb*) height were also measured with a Vertex IV ultrasonic hypsometer and the crown radii in the eight sub-cardinal directions were measured via the vertical sighting method as proposed by Preuhsler^53^.

Moreover, two increment cores were taken from each tree using an increment borer with an inner diameter of 5 mm. Coring was done in North and East direction whenever possible; the minimum requirement was an angle of 90° between both coring directions in order to minimize the error rate associated with a possible non-concentric growth. Afterwards, the cores were polished on a sanding machine with sandpaper of a coarseness ranging from 120 to 1200 grit, depending on the tree species, in order to achieve optimum visibility of the growth rings. This preparation allowed tree-ring width measurements with a precision of 0.01 mm using a Digital Positiometer after Johann^54^. Crossdating was based on the methods provided with the dplR library^55^ of the statistical programming language R version 3.2.2^56^.

From the increment cores we could trace the stem diameter growth of each tree over at least several decades on an annual basis, resulting in a total of 73,685 observations. These diameter growth series could easily be transformed into the growth of basal area which was the goal variable of our subsequent analyses. We preferred basal area instead of diameter because, in contrast to the latter, it is more directly related to a tree’s biological production. Individual tree ages could be reliably estimated by combining city administration records with increment core series.

The core structure of our statistical analyses was an equation which presumes a linear relationship between the natural logarithm of a tree’s basal area *ba* and its age

1 $$\mathrm{ln}(ba)=a+b\,\cdot \,\mathrm{ln}(age)$$

where *a* and *b* are constants, a being the intercept and b being the slope of the line described by Equation 1 in a double logarithmic coordinate system. While this linear form is convenient for statistical fitting, it translates into the following non-linear basal area growth equation:

2 $${\rm{ba}}={e}^{a}\cdot ag{e}^{b}$$

Visual data inspection showed that the basal area growth curve patterns (convex for 0 < *b* < 1, concave for *b* > 1) that can be expressed by this equation are suitable for describing the observed basal area growth.

Our first goal was to scrutinize whether, across all cities, the parameters *a* and *b* in Equations 1 and 2 changed due to recent growth trends, and whether they depend on the urban zone in which a tree is growing. In order to differentiate between the two growth-trend relevant periods, we introduced the dummy variable *recent* which is 1 for each observation later than 1959 and 0 otherwise. This is in accordance with growth trends since about the 1960s that have been identified for forest trees^5^. The urban zone affiliation (comprising trees in the original urban and suburban categories as stated above) was described by the variable *urb* which is also a dummy variable with *urb * = 1 representing urban and *urb* = 0 representing rural trees. For the related statistical analysis we designed two linear mixed models:

3 $$\mathrm{ln}(b{a}_{ijk})={a}_{0}+{a}_{1}\,\cdot \,{recen}{{t}}_{ijk}+({b}_{0}+{b}_{1}\cdot recen{t}_{ijk})\,\cdot \,\mathrm{log}(ag{e}_{ijk})+{c}_{i}\,\cdot \,\mathrm{log}(ag{e}_{ijk})+{d}_{i}+{d}_{ij}+{\varepsilon }_{ijk}$$

4 $$\mathrm{ln}(b{a}_{ijk})={a}_{0}+{a}_{1}\cdot ur{b}_{ij}+({b}_{0}+{b}_{1}\cdot ur{b}_{ij})\,\cdot \,\mathrm{log}(ag{e}_{ijk})+{c}_{i}\cdot \,\mathrm{log}(ag{e}_{ijk})+{d}_{i}+{d}_{ij}+{\varepsilon }_{ijk}$$

The first model (Equation 3) tests for an overall growth trend, the second one (Equation 4) tests for an overall urban zone effect. In both models, the indexes *i*, *j*, *k* represent the city, tree, and observation, respectively. The parameters *a*_0_, *a*_1_, *b*_0_, *b*_1_ are the fixed effects, whereby the “a” parameters are components of the intercept in Equation 1, and the “b” parameters are components of the slope, respectively. If *a*_1_ in Equation 3 differed significantly from 0, this would mean that the age-basal area relationship before 1960 had a different intercept than since. In Equation 4, this would indicate that, in general, the intercept of urban trees is not the same as for rural trees. The parameter *b*_1_ in both equations has an analogous meaning for the slope.

The “c” and “d” parameters are random effects, which are assumed to be normally distributed with the expectation of 0 (${c}_{i} \sim N(0;{\tau }_{1}^{2})$, ${d}_{i} \sim N(0;{\tau }_{2}^{2})$, ${d}_{ij} \sim N(0;{\tau }_{3}^{2})$). These random effects cover statistical dependencies which are due to the nested data structure. The random effect *c*_*i*_ covers city (and as such species) specific deviations from the general slope, while *d*_*i*_ and *d*_*ij*_ represent city, and tree-in-city specific deviations from the general intercept. The errors *ε*_*ijk*_ are assumed to be i.i.d. distributed, and ${\varepsilon }_{ijk} \sim N(0;{\sigma }^{2})$.

Our second goal was to gain more detailed insights into the combined effects of urban zone affiliation and possible period-specific growth trends, while also considering a possible differentiation across climate zones. Thus, we defined another dummy variable, *czone* which distinguished, corresponding to the four climate zones covered by the data (Table 1), the four levels *temp* (temperate climate), *bor* (boreal climate), *med* (Mediterranean climate), and *sub* (subtropical climate), with *temp* being the reference level. Introducing these three explanatory variables into Equation 1, we formulated the following mixed linear regression model, containing all possible main effects and interactions between the explanatory variables *urb*, *recent*, *czone*, and ln(*age*):

5 $$\begin{array}{ccc}{\rm{l}}{\rm{n}}(b{a}_{ijk}) & = & {a}_{0}+{a}_{1}\cdot ur{b}_{ij}+{a}_{2}\cdot czone\,bo{r}_{i}+{a}_{3}\cdot czone\,me{d}_{i}+{a}_{4}\cdot czone\,su{b}_{i}\\  &  & +\,{a}_{5}\cdot recen{t}_{ijk}+{a}_{6}\cdot ur{b}_{ij}\cdot czone\,bo{r}_{i}+{a}_{7}\cdot ur{b}_{ij}\cdot czone\,me{d}_{i}\\  &  & +\,{a}_{8}\cdot ur{b}_{ij}\cdot czone\,su{b}_{i}+{a}_{9}\cdot ur{b}_{ij}\cdot recen{t}_{ijk}+{a}_{10}\cdot czone\,bo{r}_{i}\cdot recen{t}_{ijk}\\  &  & +\,{a}_{11}\cdot czone\,me{d}_{i}\cdot recen{t}_{ijk}+{a}_{12}\cdot czone\,su{b}_{i}\cdot recen{t}_{ijk}\\  &  & +\,{a}_{13}\cdot ur{b}_{ij}\cdot czone\,bo{r}_{i}\cdot recen{t}_{ijk}+{a}_{14}\cdot ur{b}_{ij}\cdot czone\,me{d}_{i}\cdot recen{t}_{ijk}\\  &  & +\,{a}_{15}\cdot ur{b}_{ij}\cdot czone\,su{b}_{i}\cdot recen{t}_{ijk}\\  &  & +\,{\rm{l}}{\rm{n}}(ag{e}_{ijk})\cdot ({b}_{0}+{b}_{1}\cdot ur{b}_{ij}+{b}_{2}\cdot czone\,bo{r}_{i}+{b}_{3}\cdot czone\,me{d}_{i}\\  &  & +\,{b}_{4}\cdot czone\,su{b}_{i}+{b}_{5}\cdot recen{t}_{ijk}+{b}_{6}\cdot ur{b}_{ij}\cdot czone\,bo{r}_{i}\\  &  & +\,{b}_{7}\cdot ur{b}_{ij}\cdot czone\,me{d}_{i}+{b}_{8}\cdot ur{b}_{ij}\cdot czone\,su{b}_{i}+{b}_{9}\cdot ur{b}_{ij}\cdot recen{t}_{ijk}\\  &  & +\,{b}_{10}\cdot czone\,bo{r}_{i}\cdot recen{t}_{ijk}+{b}_{11}\cdot czone\,me{d}_{i}\cdot recen{t}_{ijk}\\  &  & +\,{b}_{12}\cdot czone\,su{b}_{i}\cdot recen{t}_{ijk}+{b}_{13}\cdot ur{b}_{ij}\cdot czone\,bo{r}_{i}\cdot recen{t}_{ijk}\\  &  & +\,{b}_{14}\cdot ur{b}_{ij}\cdot czone\,me{d}_{i}\cdot recen{t}_{ijk}+{b}_{15}\cdot ur{b}_{ij}\cdot czone\,su{b}_{i}\cdot recen{t}_{ijk})\\  &  & +\,{c}_{i}\,\cdot \,{\rm{l}}{\rm{n}}(ag{e}_{ijk})+{d}_{i}+{d}_{ij}+{\varepsilon }_{ijk}\end{array}$$

After fitting, this model allowed us to calculate estimates for the parameters *a* and *b* corresponding to Equation 1 for any combination of climate zone (*czone*), urbanity (*urb*) and time period (*recent*) simply by summing up the required subsets of the parameter estimates *a*_0_, *a*_1_, …, a_15_ (for the intercept) and *b*_0_, *b*_1_, …, *b*_15_ (for the slope), respectively. As the standard errors of these estimates are also known, it is possible to test any parameter combination of interest against any other. For example, the estimated intercept for an urban tree in the boreal climate zone in the time period since 1960 results from ${a}^{\ast }={a}_{0}+{a}_{1}+{a}_{2}+{a}_{5}+{a}_{6}+{a}_{9}+{a}_{10}+{a}_{13}$. Analogously, the intercept for an urban tree in the boreal zone, but in the period before 1960 is ${a}^{\ast \ast }={a}_{0}+{a}_{1}+{a}_{2}+{a}_{6}.$ Evidently the corresponding slopes must be calculated as ${b}^{\ast }={b}_{0}+{b}_{1}+{b}_{2}+{b}_{5}+{b}_{6}+{b}_{9}+{b}_{10}+{b}_{13}$, and ${b}^{\ast \ast }={b}_{0}+{b}_{1}+{b}_{2}+{b}_{6}$, respectively. Testing the null hypothesis *H*_0_: *a* ^*^ − *a* ^**^ = 0 shows whether the intercepts of the two age-basal area lines representing the conditions to be compared differ significantly or not. In the same way, the null hypothesis *H*_0_: *b* ^*^ − *b* ^**^ = 0 allows testing for significant differences between the two slopes.

Following the procedure shown above, we tested within each of the four climate zones whether slopes and intercepts differed i) between urban and rural trees before 1960, ii) between urban trees before and since 1960, iii) between rural trees before and since 1960, and iv) between urban and rural trees since 1960. If such a pair-wise comparison yielded at least one significant *p* < 0.05 difference in either the intercept or the slope, we considered the corresponding allometric lines to be different.

All statistical evaluations were conducted with the software R 3.2.2^56^, mixed model fits and parameter tests were accomplished with the function lmer from the R package lme4^57^ and the package lmerTest^58^. The post-hoc comparisons of coefficient combinations were achieved with the function glht from the package multcomp^59^.

## Electronic supplementary material

Supplementary material
