# Supplementary material for: Climate change accelerates growth of urban trees in metropolises worldwide
Source: Sci Rep. 2017 Nov 13;7:15403. doi: 10.1038/s41598-017-14831-w (PMC5684322; doi:10.1038/s41598-017-14831-w)
Supplement: Supplementary file 1 — Supplementary material [file 41598_2017_14831_MOESM1_ESM.doc]

Supplementary Material

Title: Climate change accelerates growth of urban trees in metropolises worldwide

Hans Pretzsch, Peter Biber, Enno Uhl, Jens Dahlhausen, Gerhard Schütze, Diana Perkins, Thomas Rötzer, Juan Caldentey, Takayoshi Koike, Tran van Con, Aurélia Chavanne, Ben du Toit, Keith Foster, Barry Lefer.

correspondence to: [Hans.Pretzsch@lrz.tum.de](mailto:Hans.Pretzsch@lrz.tum.de)

**
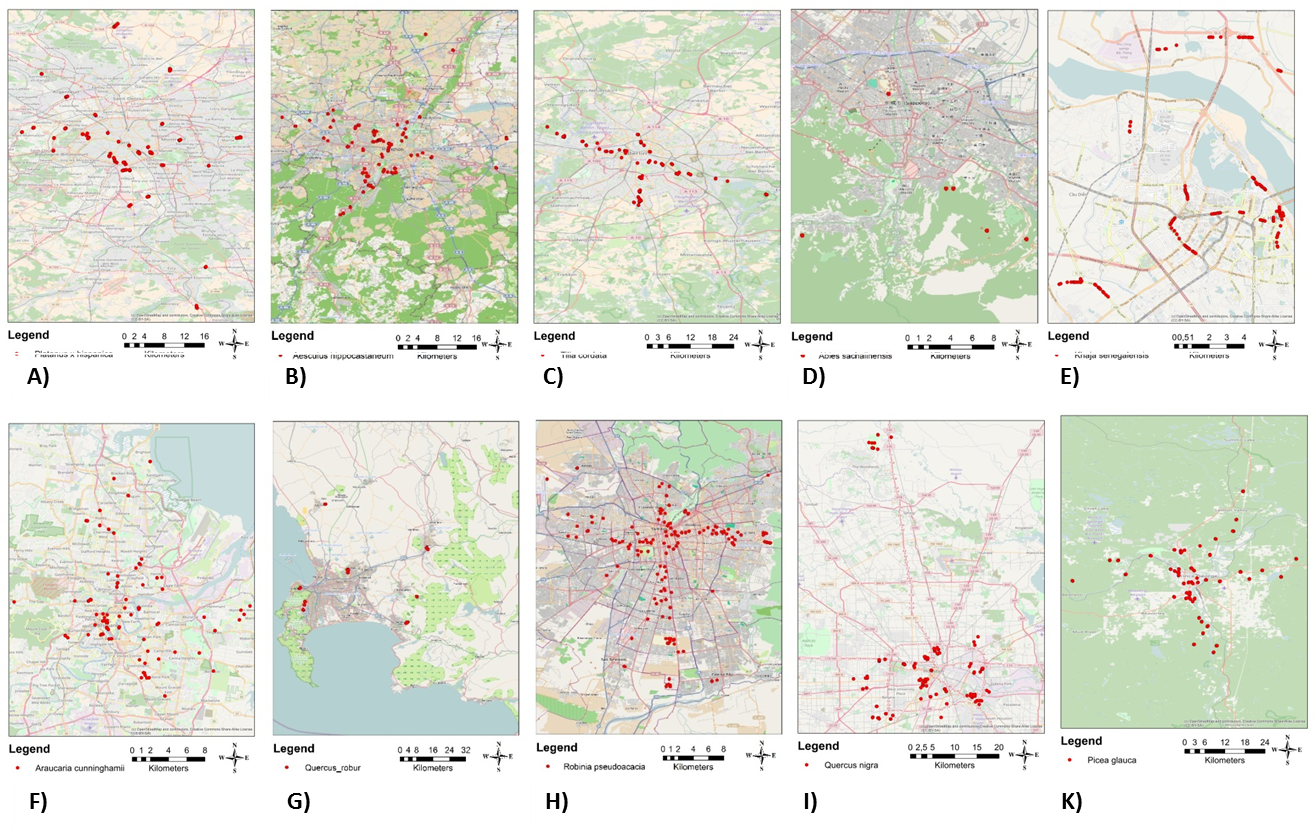
**

**Fig. S1:** City-specific sampling arrangements. Red dots indicate locations where one or more trees were sampled. A) Paris, France, B) Munich and C) Berlin, Germany, D) Sapporo, Japan, E) Hanoi, Vietnam, F) Brisbane, Australia, G) Cape Town, South Africa, H) Santiago de Chile, Chile, I) Houston, United States, K) Prince George, Canada. (Maps were created by using ArcGIS, ESRI, Version 10.1 and Open Street Maps [www.openstreetmap.org/copyright]).

**Table S1**: Results of fitting the linear mixed model described by equation 3 (n = 73,685). Significant parameter estimates are printed in bold.

| **Fixed Effects** | | | | |  | **Random Effects and Residuals** | |
| --- | --- | --- | --- | --- | --- | --- | --- |
| **Parameter** | **Variable** | **Estimate** | **Standard Error** | **p** |  | **Variable** | **Variance** |
| *a_0_* | *intercept* | **-7.8205** | 0.7147 | 0.0000 |  | *c_i_* | 0.2506 |
| *a_1_* | *recent* | **-0.4503** | 0.0174 | 0.0000 |  | *d_i_* | 5.1016 |
| *b_0_* | *log(age)* | **1.4845** | 0.1584 | 0.0000 |  | *d_ij_* | 0.4408 |
| *b_1_* | *log(age)*recent* | **0.1208** | 0.0042 | 0.0000 |  | *ε_ijk_* | 0.0774 |

**Table S2**: Results of fitting the linear mixed model described by equation 4 (n = 73,685). Significant parameter estimates are printed in bold.

| **Fixed Effects** | | | | |  | **Random Effects and Residuals** | |
| --- | --- | --- | --- | --- | --- | --- | --- |
| **Parameter** | **Variable** | **Estimate** | **Standard Error** | **P** |  | **Variable** | **Variance** |
| *a_0_* | *intercept* | **-8.6024** | 0.7210 | 0.0000 |  | *c_i_* | 0.2535 |
| *a_1_* | *urb* | **0.5783** | 0.0445 | 0.0000 |  | *d_i_* | 5.1841 |
| *b_0_* | *log(age)* | **1.6486** | 0.1593 | 0.0000 |  | *d_ij_* | 0.4300 |
| *b_1_* | *log(age)*urb* | **-0.0899** | 0.0050 | 0.0000 |  | *ε_ijk_* | 0.0780 |

**Table S3**: Results of fitting the linear mixed model described by equation 5 (n = 73,685). Significant parameter estimates are printed in bold.

| **Fixed Effects** | | | | |
| --- | --- | --- | --- | --- |
| **Parameter** | **Variable** | **Estimate** | **Standard Error** | **p** |
| *a_0_* | *Intercept* | **-7.5110** | 0.8006 | 0.0000 |
| *a_1_* | *Urb* | **0.4016** | 0.0774 | 0.0000 |
| *a_2_* | *czone bor* | **-3.8004** | 1.2664 | 0.0137 |
| *a_3_* | *czone med* | 0.9272 | 1.2664 | 0.4813 |
| *a_4_* | *czone sub* | 0.1110 | 1.1569 | 0.9253 |
| *a_5_* | *Recent* | **-0.8266** | 0.0498 | 0.0000 |
| *a_6_* | *urb * czone bor* | -0.2316 | 0.1348 | 0.0860 |
| *a_7_* | *urb * czone med* | -0.1938 | 0.1666 | 0.2448 |
| *a_8_* | *urb * czone sub* | 0.2863 | 0.2723 | 0.2932 |
| *a_9_* | *urb * recent* | **0.7075** | 0.0560 | 0.0000 |
| *a_10_* | *czone bor * recent* | **-0.2125** | 0.0865 | 0.0140 |
| *a_11_* | *czone med * recent* | **0.2761** | 0.0876 | 0.0016 |
| *a_12_* | *czone sub * recent* | -0.0283 | 0.2449 | 0.9082 |
| *a_13_* | *urb * czone bor* recent* | **-0.3510** | 0.0959 | 0.0003 |
| *a_14_* | *urb * czone med * recent* | **-0.7665** | 0.1175 | 0.0000 |
| *a_15_* | *urb * czone sub * recent* | -0.2965 | 0.2588 | 0.2518 |
| *b_0_* | *log(age)* | **1.3522** | 0.1980 | 0.0000 |
| *b_1_* | *log(age) * urb* | **-0.1149** | 0.0127 | 0.0000 |
| *b_2_* | *log(age) * czone bor* | 0.6731 | 0.3130 | 0.0615 |
| *b_3_* | *log(age) * czone med* | -0.1507 | 0.3131 | 0.6424 |
| *b_4_* | *log(age) * czone sub* | -0.0011 | 0.2903 | 0.9970 |
| *b_5_* | *log(age) * recent* | **0.1980** | 0.0125 | 0.0000 |
| *b_6_* | *log(age) * urb * czone bor* | **0.2955** | 0.0194 | 0.0000 |
| *b_7_* | *log(age) * urb * czone med* | **0.1168** | 0.0312 | 0.0002 |
| *b_8_* | *log(age) * urb * czone sub* | 0.1280 | 0.0815 | 0.1166 |
| *b_9_* | *log(age) * urb * recent* | **-0.1554** | 0.0138 | 0.0000 |
| *b_10_* | *log(age) * czone bor * recent* | **0.1116** | 0.0213 | 0.0000 |
| *b_11_* | *log(age) * czone med * recent* | **-0.0596** | 0.0214 | 0.0055 |
| *b_12_* | *log(age) * czone sub * recent* | **0.1764** | 0.0784 | 0.0244 |
| *b_13_* | *log(age) * urb * czone bor * recent* | 0.0470 | 0.0240 | 0.0502 |
| *b_14_* | *log(age) * urb * czone med * recent* | **0.1798** | 0.0299 | 0.0000 |
| *b_15_* | *log(age) * urb * czone sub * recent* | -0.1132 | 0.0818 | 0.1664 |
|  |  |  |  |  |
| **Random Effects and Residuals** | | | | |
| **Variable** | | **Variance** | | |
| *c_i_* | | 0.1172 | | |
| *d_i_* | | 1.9081 | | |
| *d_ij_* | | 0.4131 | | |
| *ε_ijk_* | | 0.0755 | | |

**Table S4 A**: Test results for deviations from the model predictions presented in Figure 3A (boreal climate zone). The parameter estimates given in Table S3 allow calculation of the intercept *a* and the slope *b* as denoted in equation 1 for any desired combination of the predictor variables *czone* (climate zone), *urb* (urban vs. rural), and *recent* (period before 1960 vs. period since 1960). The table shows the resulting intercept and slope values and the result of testing for significant differences (***: p < 0.001, n.s.: not significant). Gray-shaded fields indicate combinations which were not tested because they were not meaningful.

| Intercept boreal | | | rural | urban | |
| --- | --- | --- | --- | --- | --- |
|  |  |  | since 1960 | before 1960 | since 1960 |
|  |  | value | -12.3505 | -11.1413 | -11.8239 |
| rural | before 1960 | -11.3114 | *** | n.s. |  |
|  | since 1960 | -12.3505 |  |  | n.s. |
| urban | before 1960 | -11.1413 |  |  | n.s. |

| Slope boreal | | | rural | urban | |
| --- | --- | --- | --- | --- | --- |
|  |  |  | since 1960 | before 1960 | since 1960 |
|  |  | value | 2.3349 | 2.2059 | 2.4071 |
| rural | before 1960 | 2.0253 | *** | *** |  |
|  | since 1960 | 2.3349 |  |  | *** |
| urban | before 1960 | 2.2059 |  |  | *** |

**Table S4 B**: Test results for deviations from the model predictions presented in Figure 3B (temperate climate zone). See Table S4 A for more details. Gray-shaded fields indicate combinations which were not tested because they were not meaningful (***: p < 0.001).

| Intercept temperate | | | rural | urban | |
| --- | --- | --- | --- | --- | --- |
|  |  |  | since 1960 | before 1960 | since 1960 |
|  |  | value | -8.3376 | -7.1093 | -7.2284 |
| rural | before 1960 | -7.5110 | *** | *** |  |
|  | since 1960 | -8.3376 |  |  | *** |
| urban | before 1960 | -7.1093 |  |  | *** |

| Slope temperate | | | rural | urban | |
| --- | --- | --- | --- | --- | --- |
|  |  |  | since 1960 | before 1960 | since 1960 |
|  |  | value | 1.5502 | 1.2373 | 1.2799 |
| rural | before 1960 | 1.3522 | *** | *** |  |
|  | since 1960 | 1.5502 |  |  | *** |
| urban | before 1960 | 1.2373 |  |  | *** |

**Table S4 C**: Test results for deviations from the model predictions presented in Figure 3C (Mediterranean climate zone). See Table S4 A for more details. Gray-shaded fields indicate combinations which were not tested because they were not meaningful (***: p < 0.001, n.s.: not significant).

| Intercept Mediterranean | | | rural | urban | |
| --- | --- | --- | --- | --- | --- |
|  |  |  | since 1960 | before 1960 | since 1960 |
|  |  | Value | -7.1342 | -6.3759 | -6.9854 |
| rural | before 1960 | -6.5837 | *** | n.s. |  |
|  | since 1960 | -7.1342 |  |  | n.s. |
| urban | before 1960 | -6.3759 |  |  | *** |

| Slope Mediterranean | | | rural | urban | |
| --- | --- | --- | --- | --- | --- |
|  |  |  | since 1960 | before 1960 | since 1960 |
|  |  | Value | 1.3400 | 1.2034 | 1.3663 |
| rural | before 1960 | 1.2015 | *** | n.s. |  |
|  | since 1960 | 1.3400 |  |  | n.s. |
| urban | before 1960 | 1.2034 |  |  | *** |

**Table S4 D**: Test results for deviations from the model predictions presented in Figure 3D (subtropical climate zone). See Table S4 A for more details. Gray-shaded fields indicate combinations which were not tested because they were not meaningful (**: p < 0.01, ***: p < 0.001, n.s.: not significant).

| Intercept subtropical | | | rural | urban | |
| --- | --- | --- | --- | --- | --- |
|  |  |  | since 1960 | before 1960 | since 1960 |
|  |  | value | -8.2548 | -6.7120 | -7.1559 |
| rural | before 1960 | -7.3400 | *** | ** |  |
|  | since 1960 | -8.2548 |  |  | *** |
| urban | before 1960 | -6.7120 |  |  | *** |

| Slope subtropical | | | rural | urban | |
| --- | --- | --- | --- | --- | --- |
|  |  |  | since 1960 | before 1960 | since 1960 |
|  |  | value | 1.7255 | 1.3641 | 1.4699 |
| rural | before 1960 | 1.3511 | *** | n.s. |  |
|  | since 1960 | 1.7255 |  |  | *** |
| urban | before 1960 | 1.3641 |  |  | *** |
